# Exploring the roles of fear and powerlessness in the relationship between perceived risk of the COVID-19 pandemic and information-avoidance behavior

**DOI:** 10.3389/fpsyg.2022.1005142

**Published:** 2022-11-15

**Authors:** Kunying Zhang, Naifu Zhang, Jie Wang, Jie Jiang, Sihua Xu

**Affiliations:** ^1^School of Education, Huaibei Normal University, Huaibei, China; ^2^School of Education, Hangzhou Normal University, Hangzhou, China; ^3^College of International Business, Shanghai International Studies University, Shanghai, China; ^4^The Second Affiliated Hospital of Anhui University of Traditional Chinese Medicine, Hefei, China

**Keywords:** fear, powerlessness, perceived epidemic risk, information-avoidance behavior, COVID-19

## Abstract

The COVID-19 has seriously impacted various aspects of the society on a global scale. However, it is still unclear how perceived risk influences epidemic information-avoidance behavior which generally helps us understand public information avoidance. This study aimed to assess the relationship between the perceived epidemic risk and information-avoidance behavior and the mediating role of fear and powerlessness during the COVID-19 pandemic in China. A total of 557 Chinese respondents with COVID-19 treated in modular hospitals ranging from 16 to 72 years old were recruited and completed questionnaires in the face-to-face manner containing scales of the perceived epidemic risk of COVID-19, fear, powerlessness, and information-avoidance behavior. To test the conceptual model, we adopted structural equation modeling (SEM) with the perceived risk of the COVID-19 pandemic as a predictor, fear and powerlessness as mediating variables, and information-avoidance behavior as the outcome. The results indicated a significant and positive association between the perceived epidemic risk of COVID-19 and information-avoidance behavior. Powerlessness acted as the mediator between the perceived epidemic risk of COVID-19 and information-avoidance behavior. The perceived epidemic risk of COVID-19 influenced information-avoidance behavior through fear and powerlessness in turn. Findings from this study implied that public health managers should consider the mediating roles of negative emotions such as fear and powerlessness for coping with behaviors in public health emergencies, especially the information avoidance behaviors related to risk perception.

## Introduction

The new coronavirus disease named coronavirus disease 2019 (COVID-19) by the World Health Organization has spread around the world and become a public health concern due to its high level of contagion (Guan et al., 2020). The vulnerability of COVID-19 has made many people become aware that they are at constant risk of infection, which influences their coping behavior and decisions (Rana et al., 2021). When facing a perceived epidemic risk, the first thing that most people think about is how to avoid risk-related outcomes, which further motivates mental and behavioral responses to the looming risk. Seeking and avoiding additional information related to this risk are two specific behavioral reactions to risky situations (Gutteling and De Vries, 2017).

Information-seeking behavior related to the perceived risk of COVID-19 can be defined as an active or purposeful behavior undertaken by an individual to find information about COVID-19 (Zimmerman and Shaw, 2020). The protection motivation theory regarded information behavior as a response to fear caused by potential threats, and posited that messages related to a certain risk or threat stimulated people to assess the severity of a risky situation and the capability of their response to the situation to protect themselves (Rogers, 1975). Previous studies have also demonstrated a positive relationship between individuals’ risk perception and information-seeking behavior (Gutteling and De Vries, 2017; Huang and Yang, 2020; Wen, 2020).

Although information related to a risk is very important and valuable to protect against threats, people do not always seek it and sometimes spare no effort to avoid it (Sweeny and Miller, 2012; Emanuel et al., 2015). Namely, information-avoidance behavior can be defined as any behavior that intended to prevent or delay the acquisition of available but potentially unwanted information to cope with perceived risk during a pandemic (Sweeny et al., 2010; Demetriades and Walter, 2016). Information-avoidance behavior also plays an important role in information management when coping with perceived risk in a health context (Liu et al., 2021). On the one hand, information-avoidance behavior can serve as a strategy to control negative emotions and maintain optimism and hope (Barbour et al., 2012). On the other hand, information-avoidance behavior can also make individuals being unable to obtain epidemic-related information from a disease control standpoint, thus increased the unmitigated spread of disease. However, information avoidance has received less attention than information seeking, and the relationship between information-avoidance behavior and risk perception is still controversial (Yang et al., 2021). Some researchers have revealed that risk perception positively predicted information-avoidance behaviors (Witte, 1996; Taber et al., 2015). Other researchers have found that risk perception negatively predicted information avoidance (Yang and Kahlor, 2013). This literature suggested that the relationship between risk perception and information avoidance was more complex. Therefore, it is necessary to further explore the relationship between these factors and related psychological paths in the middle of the COVID-19 pandemic. Hence, the Hypothesis 1 in the present study was proposed as follows.

*Hypothesis 1*: Individuals’ perceived risk during COVID-19 was correlated with information-avoidance behaviors.

When discussing the relationship between risk perception and information avoidance, it is hard to discuss it alone without considering affect or emotion, because they often rely on and influence each other in empirical research (Griffin et al., 1999). The Planned Risk Information Avoidance (PRIA) Model proposed by Deline and Kahlor (2019) also believed that affective components (such as emotional valence) were important predictors of an individual’s information-avoidance behavior. Previous research on the relationship between risk perception and information avoidance reported that the risk perception’s impact on information avoidance was mediated by affective responses such as concern, worry, and anxiety (Yang and Kahlor, 2013; Kahlor et al., 2020). As a major psychological reaction to health crises, fear has also often been discussed in previous studies (Dunwoody and Griffin, 2015; Emanuel et al., 2015; Petzold et al., 2020). In general, fear has been regarded as a negative emotion derived from perceived threats to an individual’s well-being, and was characterized by increased arousal, behavioral tendencies, and negative apprehensions (Dillard et al., 2021; Mertens et al., 2021). When individuals recognized that their environment contained a risk to which they were susceptible and that risk worked against their goal of maintaining their well-being, they tended to experience fear (Lazarus, 1991). Moreover, people have been affected by stressful experiences during the COVID-19 pandemic, and their most common response to the outbreak of COVID-19 was fear (Porcelli, 2020). For example, in a recent study recruiting 6,509 participants in Germany, Petzold et al. (2020) found that approximately 45% of the participants reported fear of being infected with COVID-19. Assuming that people were aware of the pandemic and the elevated risk during the crisis of the COVID-19, individuals with higher risk perception may perceive the pandemic to be a more significant threat to their well-being, which may cause stronger fear. Consistent with this inference, empirical evidence has revealed a positive relationship between the perceived epidemic risk and their fear during COVID-19 (Harper et al., 2021). Regarding the correlation between perceived epidemic risk or fear and information-avoidance behavior, Dunwoody and Griffin (2015) assumed that perceived epidemic risk triggered information avoidance when this risk made an individual too fearful. However, Emanuel et al. (2015) found that information avoidance behavior was unrelated to perceived epidemic risk. Therefore, it remains unclear whether perceived epidemic risk triggers information-avoidance behavior through fear in the middle of the COVID-19 pandemic. Hence, the Hypothesis 2 in the present study was proposed as follows.

*Hypothesis 2*: Fear mediated the relationship between the individuals’ perceived risk during COVID-19 and information-avoidance behaviors.

In addition to fear as a discrete emotion, we supplemented the model with feelings of powerlessness in the face of epidemic risk. Powerlessness has been regarded as the psychological experience in which an individual felt out of control and unable to cope with current or future events (Braga and Cruz, 2009). It was also a common psychological response to the outbreak of COVID-19 in China (Guo et al., 2020; Liu et al., 2021). For example, Liu et al. (2021) conducted a survey on the mental health of Wuhan citizens during the COVID-19 pandemic, and further demonstrated that the residents of Wuhan experienced various levels of powerlessness. Recent studies reported that individuals in a lasting crisis event who felt higher level of perceived epidemic risk during COVID-19 tended to use less social media, and showed more information-avoidance behavior (Ravindran et al., 2014; Chen et al., 2021). Importantly, a more recent cross-sectional survey on the psychosocial impact of COVID-19 revealed that fear was a predictor of powerlessness (Jin and Ryu, 2022). Based on these findings, powerlessness may play an important role in the perceived epidemic risk of COVID-19-triggered information-avoidance behavior. Hence, the Hypothesis 3 and Hypothesis 4 in the present study were proposed as follows.

*Hypothesis 3*: Powerlessness mediated the relationship between the individuals’ perceived risk during COVID-19 and information-avoidance behaviors.

*Hypothesis 4*: Fear and powerlessness serially mediated the relationship between the individuals’ perceived risk during COVID-19 and information-avoidance behaviors.

Overall, previous studies have found that individuals exhibited emotional reactions such as powerlessness and fear during the COVID-19 pandemic, and have also revealed that the perceived epidemic risk of COVID-19 was associated with information-avoidance behavior. However, whether the perceived epidemic risk of COVID-19 predicts information-avoidance behavior is still controversial. Importantly, the psychological path of perceived epidemic risk of COVID-19 on information-avoidance behavior remains unclear. To fill this gap, the present study was performed to investigate the complex associations among those variables using a structural equation modeling (SEM) approach.

## Materials and methods

### Participants and procedures

Given that convenience sampling has been considered as a specific type of random sampling method and provided the highest response level while saving resources and time (Jager et al., 2017), Chinese respondents with COVID-19 treated in modular hospitals in Shanghai were randomly recruited and completed the survey in the face-to-face manner. The total sample size was 557, and all were included in the analysis. As shown in Table 1, the sample was predominantly male (75.8%). Approximately 28% of respondents were 40–49 years old, 23.5% were 30–39 years old, 21% were 20–29 years old, and 19.7% were 50–59 years old. The education level of respondents ranged from “less than high school” to “graduate degree.” This survey was approved by the Local Ethics Committee and conformed to the principles of the Declaration of Helsinki, and included items concerning risk perception, feelings of fear and powerlessness, and information avoidance. The respondents filled out the informed consent document and were told that the purpose of this survey was only for research and their participation was voluntary and completely anonymous, apart from certain demographic data.

**Table 1:** Descriptive statistics analysis of demographic information.

Demographics		Percentage (%)
Gender	Male	75.8
	Female	24.2
Age	<20 years	3.2
	20–29 years	21
	30–39 years	23.5
	40–49 years	28
	50–59 years	19.7
	60–69 years	4.3
	≥70 years	0.3
Education	Less than high school	48.7
	High school graduate	32.1
	Undergraduate	9.5
	Graduate and above	9.7

The data-gathering phase started from April 12, 2022, to May 5, 2022. Anderson and Gerbing (1988) advised that a small sample size from 100 to 150 was adequate for a simple structural model, and a more complex structural model requires a larger sample size. The sample sizes of 366, and 374 have been employed in previous studies for testing study models of 4–6 hypotheses (Sumarliah et al., 2021a, b). According to Fugard and Potts (2015), the present study adopted the formula (*n* = $\frac{z^{2}\times p\times q}{e^{2}}$ ) with identical parameters to determine the adequate sample size (Fugard and Potts, 2015; Wang et al., 2022). Based on this equation, it can be determined that the adequate sample size for this study is 384. Thus, the sample size of 577 in this study is adequate to test our hypothesis of the framework.

### Measures

The present study followed a cross-sectional design and used a face-to-face questionnaire as the data collection method. To assess the perceived epidemic risk of COVID-19, fear, powerlessness, and information-avoidance behavior of those infected with COVID-19, we adopted the scales used in the previous literature. See Table 2 for complete item details.

**Table 2:** Summary of the dependent, independent, and mediating variables.

Variable	Wording	*M*	SD
Perceived epidemic risk scale	Average index	3.04	0.76
(ω = 0.886)	I think COVID-19 is very contagious	2.87	1.00
	I think that even with proper protection, there is still a risk of infection with COVID-19	2.76	0.90
	I am still not very clear about the government’s measures to control COVID-19	2.89	0.97
	I do not know what caused COVID-19	3.06	0.93
	I think there is still a risk of infection after COVID-19 is cured	3.24	0.95
	After COVID-19 is cured, I think it may still have an impact on the body	3.40	0.94
Fear scale		3.03	0.80
(ω = 0.916)	I am most afraid of COVID-19	3.15	0.93
	It makes me uncomfortable to think about COVID-19	3.06	0.87
	I worry a lot about COVID-19	2.89	0.93
	I am afraid of losing my life because of COVID-19	2.96	0.92
	My hands become clammy when I think about COVID-19	3.10	0.97
Powerlessness scale		2.97	0.75
(ω = 0.762)	I felt incapable of looking after myself after I was infected with COVID-19	2.83	0.82
	I felt I could do nothing after I was infected with COVID-19	3.11	0.85
Information-avoidance scale		2.34	0.77
(ω = 0.791)	I did not want any more information about COVID-19 after I was infected	2.42	0.87
	I intentionally ignored some information related to COVID-19 after I was infected	2.26	0.81

The perceived epidemic risk for the respondents infected with COVID-19 was measured using six items adapted from previous research (Deng and Feng, 2022). The items were scored using a Likert-type response scale ranging from 1 “strongly disagree” to 5 “strongly agree.” McDonald’s Omega value of the perceived epidemic risk scale was 0.886 in the current study.

Fear of COVID-19 was measured using five items adapted from previous studies (Doshi et al., 2021; Ahorsu et al., 2022). Respondents were asked to rate the extent to which they agreed with statements regarding the fear of COVID-19 on a Likert-type response scale ranging from 1 “strongly disagree” to 5 “strongly agree.” McDonald’s Omega value of the fear scale was 0.916 in the current study.

Powerlessness was measured using two items adapted from Braga and Cruz (2009) and Deng and Feng (2022). Respondents were asked to rate the extent to which they agreed with statements regarding their powerlessness to COVID-19 on a Likert-type response scale ranging from 1 “strongly disagree” to 5 “strongly agree.” McDonald’s Omega value of the powerlessness scale was 0.762 in the current study.

Information-avoidance behavior was measured using two items adapted from previous studies (Howell and Shepperd, 2016; Kim et al., 2020). Respondents were asked to rate the extent to which they agreed with statements regarding whether to take evasive action against information about COVID-19 on a Likert-type response scale ranging from 1 “strongly disagree” to 5 “strongly agree.” McDonald’s Omega value of the information-avoidance behavior scale was 0.791 in the current study.

### Data analysis

Data were analyzed *via* SPSS 16.0 and IBM SPSS AMOS 26. First, correlations among the study variables were determined, and the reliability of constructs was conducted in SPSS. To analyze the conceptual model, SEM was performed using AMOS 26; the perceived epidemic risk of COVID-19 was identified as a predictor, fear and powerlessness as mediators, and information-avoidance behavior as the outcome. The fitness of the model was good [χ^2^ (84) = 338.20; *p* < 0.001, RMSEA = 0.07 90% CI = (0.065, 0.082), SRMR = 0.05, NFI = 0.93, and CFI = 0.94]. A non-parametric bootstrap method (5,000 samples) was used to test the significance of the mediating effects, with a 95% CI failing to contain zero, indicating a significant mediation effect (Hu and Bentler, 1999).

## Results

### Common method biases

After data collection completion, we used Harman’s single factor test to examine possible common method bias of all variables in this study. Exploratory factor analysis was run for all items of variables with rotated principal component and four factors with eigenvalues greater than one. The first factor accounted for 32.17% of the total variance, which was below the threshold of 40%, suggesting no significant common method bias in the measurements (Podsakoff et al., 2003; Zhou and Long, 2004).

### Correlation analyses

The Pearson correlation analysis results shown in Table 3 indicated that all the scales were significantly correlated except for the correlation between the fear and information-avoidance behavior (*r* = 0.05, *p* = 0.960). The perceived epidemic risk of COVID-19 was positively and sufficiently associated with fear (*r* = 0.15, *p* < 0.001) as well as powerlessness (*r* = 0.26, p < 0.001) and information-avoidance behavior (*r* = 0.28, *p* < 0.001). Fear was positively and significantly associated with powerlessness (*r* = 0.30, *p* < 0.001), and powerlessness was positively and significantly associated with information-avoidance behavior (*r* = 0.26, *p* < 0.001).

**Table 3:** Pearson correlations for all study variables (*n* = 557).

Variable	1	2	3	4
1. Perceived risk	1			
2. Fear	0.15^***^	1		
3. Powerlessness	0.26^***^	0.30^***^	1	
4. Information-avoidance behavior	0.28^***^	0.05	0.26^***^	1

***p < 0.001.

### Relationships between the perceived epidemic risk of COVID-19 and information-avoidance behavior: Mediating effects of fear and powerlessness

**Figure 1:**
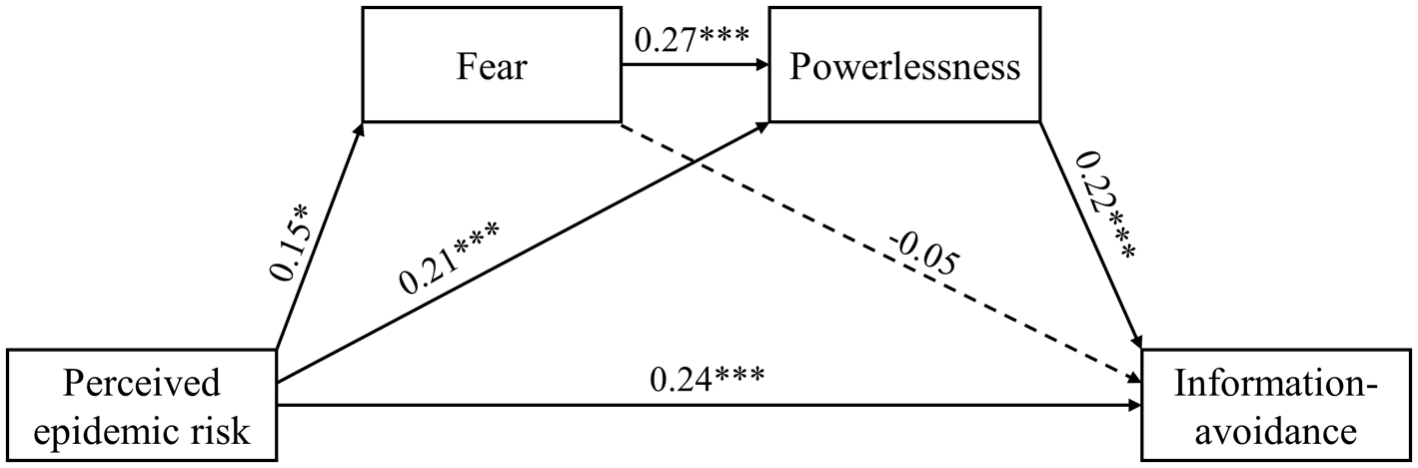
Structural equation model of the direct effect of perceived epidemic risk on other variables and the mediating effects of fear and powerlessness. The dotted lines indicate non-significant relationships. *p < 0.05, ***p < 0.001.

Based on the results of the correlation analysis and our hypothesis that fear and powerlessness mediate the relationship between the perceived epidemic risk of COVID-19 and information-avoidance behavior, we used AMOS 26.0 to test the mediating model. The results of the regression analyses are shown in Figure 1. The perceived epidemic risk of COVID-19 positively predicted information-avoidance behavior (β = 0.24, *p* < 0.001) as well as fear (β = 0.15, *p* = 0.013) and powerlessness (β = 0.21, *p* < 0.001). Fear significantly predicted powerlessness (β = 0.27, *p* < 0.001); however, fear did not predict information-avoidance behavior (β = −0.05, *p* = 0.321). A strong regression path was found between powerlessness and information-avoidance behavior (β = 0.22, *p* < 0.001).

To test the intermediary roles of fear and powerlessness in the relationship between the perceived epidemic risk of COVID-19 and information-avoidance behavior, the bootstrap method was used to sample 5,000 times and build a 95% unbiased correction CI. The results showed that the intermediary chain effect of fear and powerlessness was significant [β = 0.009, 95% CI (0.002, 0.020)], indicating significant mediation by fear and powerlessness. In addition, the perceived epidemic risk of COVID-19 had an indirect effect on information-avoidance behavior through powerlessness [β = 0.047, 95% CI (0.025, 0.079)].

## Discussion

In the present study, we first examined whether the perceived epidemic risk of COVID-19 was associated with information-avoidance behavior in patients with COVID-19. Importantly, we also aimed to establish the mediating effect of feelings of fear and powerlessness in the association between the perceived epidemic risk of COVID-19 and information-avoidance behavior.

Consistent with the Hypothesis 1, this study confirmed that the perceived epidemic risk of COVID-19 positively predicted information-avoidance behavior. This confirmed the results of previous studies (Witte, 1996; Dunwoody and Griffin, 2015; Taber et al., 2015), but was also inconsistent with a previous study suggesting that risk perception negatively predicted information-avoidance behavior (Yang and Kahlor, 2013). This contradiction may arise from different respondents and risk issues. Yang and Kahlor (2013) research was mainly concerned with the relationship between college students’ risk perception related to ecological hazards from climate change and information-avoidance behavior. Inconsistent with the previous study, this study mainly focused on the relationship between the perceived epidemic risk of COVID-19 and information-avoidance behavior in patients with COVID-19. Considering that the COVID-19 pandemic was a global public crisis and had posed a great threat to individuals, the results indicated that higher risk perception could motivate an individual to avoid information if the information was perceived as too threatening (Deline and Kahlor, 2019). This argument was consistent with the assumption that individuals avoid information to mitigate emotional burdens (van ‘t Riet and Ruiter, 2013).

In addition, we found that risk perception was directly related to negative emotions such as anger, anxiety, and stress (Griffin et al., 1999; Oh et al., 2021). This result confirmed and extended the findings in the previous studies, and demonstrated that the higher the perceived epidemic risk of COVID-19 by respondents, the more intense the experience of fear and powerlessness would be. According to the Extended Parallel Process Model, affective response to events depended on whether individuals felt that they could control the risk (Witte, 1994). Negative emotions were aroused when individuals took the risk seriously and believed that the threat would affect them personally. This means that respondents in this study felt fear and powerlessness, perhaps because they had been infected with COVID-19 and believed that their actions would not significantly affect an outcome, and lost control of the current situation during the crisis (Braga and Cruz, 2009).

Another aim of this study was to investigate the mediating effects of fear and powerlessness on the relationship between the perceived epidemic risk of COVID-19 and information-avoidance behavior. According to the protection motivation theory proposed by Rogers (1975), individuals usually used threat and coping appraisal to protect themselves in the face of threats. This view regarded information-avoidance behavior as a response to fear aroused by perceived epidemic risk. Consistent with this claim, previous studies of risk-related information behavior have demonstrated a positive relationship between perceived epidemic risk and information-avoidance behavior through feelings of fear (Jepson and Chaiken, 1990; Witte, 1994; Chiang and Tang, 2022). Notably, these studies mainly focused on the context of chronic risks for which people may not perceive a strong sense of urgency or prioritize acting immediately (Zhao and Liu, 2021). For example, Chiang and Tang (2022) mainly focused on internet security compliance behavior in a public Wi-Fi usage condition, and demonstrated that fear was an important determinant of the user’s avoidance behavior. Inconsistent with the claim of the protection motivation theory and the Hypothesis 2, feelings of fear did not mediate the association between the perceived epidemic risk of COVID-19 and information-avoidance behavior in the current study. These findings suggested that whether perceived risk affected information-avoidance behavior through fear may depend on the level of fear.

Notably, although no mediating effect of fear was observed in this sample, the SEM results corroborated the Hypothesis 3, and revealed that powerlessness mediated the relationship between the perceived epidemic risk of COVID-19 and information-avoidance behavior. In addition, the perceived epidemic risk of COVID-19 influenced information-avoidance behavior through feelings of fear and powerlessness in turn which also aligned with the Hypothesis 4. These findings confirmed and enriched one school of thought that individuals resorted to defensive avoidance to reduce their negative emotions when they felt chronic fear or when their fear has reached a certain level (Jepson and Chaiken, 1990; Witte, 1994). Namely, in the acute risk situation, individuals infected with COVID-19 exhibited a sense of powerlessness to reduce or diminish their fear of COVID-19, which stimulated information-avoidance behavior. In this case, a sense of powerlessness in coping with the perceived epidemic risk of COVID-19 could serve as a buffer to resist the negative effects of fear related to the specific threat or risk from COVID-19. Consistent with this inference, correlational evidence in the previous studies also indicated that the levels of fear were positively associated with a sense of powerlessness in the middle of the COVID-19 pandemic (Lifshin et al., 2020). In the context of COVID-19, individuals with powerlessness tended to avoid information regarding the risks when they felt a greater likelihood of being personally affected (Chen et al., 2021; Zhao and Liu, 2021).

With a view to theoretical application, this study confirmed the positive association between the perceived epidemic risk of COVID-19 and information-avoidance behaviors, and further deepened the PRIA by analyzing its mediating effect in the path of information avoidance. Compared with the PRIA in which the “affective risk response” was used as a mediating variable for risk perception to positively predict information-avoidance behavior, this study further explained the mechanism of this effect from the perspective of mediating influence. That is, when we considered the impact of specific emotional variables such as fear and powerlessness, the perceived epidemic risk of COVID-19 could not only directly affect information-avoidance behavior, but also indirectly affected the information-avoidance mechanism through fear and powerlessness in turn.

With a view to practical application and influencing of policy, the epidemic risk of COVID-19 perceived by citizens impacted their negative and information-avoidance behavior in turn. In this regard, individuals were more likely to avoid information when they felt the huge threat posed by the risk of COVID-19 and experienced negative emotions. Thus, interventions to improve risk information resources and popularize knowledge related to emotion regulation to the public may effectively reduce information-avoidance behavior among vulnerable individuals. On the one hand, the government should consider providing more positive risk information to motivate, and reassure the public by demonstrating the benefits and effectiveness of the prevention guidelines instead of simply focusing on the statistics of mortality and infection rates. On the other hand, the government should help the public to ease their negative emotions using different approaches such as social media, emotional management guidelines, and psychological counseling.

A few limitations of this study should be noted. First, although correlations between the variables were revealed in the current study, the cross-sectional design did not allow for causal conclusions in terms of the relationship analyzed. Moreover, due to the urgency of treatment of respondents who were infected with COVID-19 and insufficient manpower, we just focused on the prediction of perceived epidemic risk to information-avoidance behavior and its mediation effect, lacking justification of the respondent. Therefore, the results of this study should be confirmed by further longitudinal studies. Second, all data were collected through a survey of Chinese respondents with COVID-19 treated in modular hospitals in Shanghai, which undermined the generalization of results. Moreover, the sample in this study was quite asymmetric in relation to the level of education, which was a factor intuitively related to the individual’s ability to assimilate information from reliable sources and to interpret and value this information (Viswanath, 2005). In this case, the information-avoidance behavior may be influenced by schooling according to the level of education, even though the results did not change significantly when we carried out the analysis independently in two groups determined by schooling (low and high schooling) and the previous study demonstrated that schooling did not interfere with information-avoidance behavior (Gaspar et al., 2016). Therefore, caution should be used when extending the results to populations in other regions or other groups. Finally, the association between the perceived epidemic risk of COVID-19 and information-avoidance behavior appeared to be more complex, which may be affected by several factors, such as informational subjective norms, personal characteristics, and coping resources (Loiselle, 2019; Soroya et al., 2021). Feelings of fear and powerlessness could only explain a small part of it. We have not investigated other important factors concerning this relationship. Therefore, a more comprehensive model should be established to explore the psychological path of the association between the perceived epidemic risk of COVID-19 and information-avoidance behavior in the future.

In summary, this study has made two contributions. First, it established a relationship between the perceived epidemic risk of COVID-19 and information-avoidance behavior in the context of a public health emergency. Specifically, the perceived epidemic risk of COVID-19 could positively predict information-avoidance behavior. Second, it revealed the psychological path of perceived epidemic risk positively predicted information-avoidance behavior. Particularly, although perceived epidemic risk did not affect information-avoidance behavior alone through fear, it did affect information-avoidance behavior through fear and powerlessness in turn. In addition, perceived epidemic risk affected information-avoidance behavior through powerlessness. These findings contributed to the theoretical understanding on how the variables related to or affected each other, and further provided insights for practical implications, such as establishing programs that may decrease the fear and powerlessness in crises or emergencies to reduce information-avoidance behavior.

## Data availability statement

The raw data supporting the conclusions of this article will be made available by the authors, without undue reservation.

## Ethics statement

The studies involving human participants were reviewed and approved by Internal Review Board of the Key Laboratory of Applied Brain and Cognitive Sciences at Shanghai International Studies University. The patients/participants provided their written informed consent to participate in this study.

## Author contributions

SX conceived and designed the research, and provided critical revisions. JW, NZ, and KZ collected the data. NZ analyzed the data with feedback from SX. SX and JJ drafted the manuscript. All authors contributed to the article and approved the submitted version.

## Funding

This research was supported in part by the National Natural Science Foundation of China (72171151 and 31400872), Natural Science Foundation of Shanghai (21ZR14616), and Fundamental Research Funds for the Central Universities (2021114003).

## Conflict of interest

The authors declare that the research was conducted in the absence of any commercial or financial relationships that could be construed as a potential conflict of interest.

## Publisher’s note

All claims expressed in this article are solely those of the authors and do not necessarily represent those of their affiliated organizations, or those of the publisher, the editors and the reviewers. Any product that may be evaluated in this article, or claim that may be made by its manufacturer, is not guaranteed or endorsed by the publisher.
